# Partial Optimization of the 5-Terminal Codon Increased a Recombination Porcine Pancreatic Lipase (opPPL) Expression in *Pichia pastoris*

**DOI:** 10.1371/journal.pone.0114385

**Published:** 2014-12-29

**Authors:** Hua Zhao, Dan Chen, Jiayong Tang, Gang Jia, Dingbiao Long, Guangmang Liu, Xiaoling Chen, Haiying Shang

**Affiliations:** 1 Animal Nutrition Institute, Sichuan Agricultural University, Chengdu, Sichuan 611130, China; 2 Chongqing Academy of Animal Science, Chongqing, 402460, China; Russian Academy of Sciences, Institute for Biological Instrumentation, Russian Federation

## Abstract

Pancreatic lipase plays a key role in intestinal digestion of feed fat, and is often deficient in young animals such as weaning piglets. The objective of this study was to express and characterize a partial codon optimized porcine pancreatic lipase (opPPL). A 537 bp cDNA fragment encoding N-terminus amino acid residue of the mature porcine pancreatic lipase was synthesized according to the codon bias of *Pichia pastoris* and ligated to the full-length porcine pancreatic lipase cDNA fragment. The codon optimized PPL was cloned into the pPICZαA (Invitrogen, Beijing, China) vector. After the resultant opPPL/pPICZαΑ plasmid was transformed into *P.pastoris*, the over-expressed extracellular opPPL containing a His-tag to the C terminus was purified using Ni Sepharose affinity column (GE Healthcare, Piscataway, NJ, USA), and was characterized against the native enzyme (commercial PPL from porcine pancreas, Sigma). The opPPL exhibited a molecular mass of approximately 52 kDa, and showed optimal temperature (40°C), optimal pH (8.0), *K_m_* (0.041 mM), and *V_max_* (2.008 µmol.mg protein ^−1^.min^−1^) similar to those of the commercial enzyme with p-NPP as the substrate. The recombinant enzyme was stable at 60°C, but lost 80% (P<0.05) of its activity after exposure to heat ≥60°C for 20 min. The codon optimization increased opPPL yield for ca 4 folds (146 mg.L^−1^ vs 36 mg.L^−1^) and total enzyme activity increased about 5 folds (1900 IU.L^−1^ vs 367 IU.L^−1^) compared with those native naPPL/pPICZαΑ tranformant. Comparison of gene copies and mRNA profiles between the two strains indicated the increased rePPL yields may partly be ascribed to the increased protein translational efficiency after codon optimization. In conclusion, we successfully optimized 5-terminal of porcine pancreatic lipase encoding gene and over-expressed the gene in *P. pastoris* as an extracellular, functional enzyme. The recombination enzyme demonstrates a potential for future use as an animal feed additive for animal improvement.

## Introduction

As a family member of serine hydrolases, Lipases (EC 3.1.1.3) are a class of hydrolases that catalyse a wide range of reactions including hydrolysis, interesterification, alcholysis, acidolysis, esterification and aminolysis. They catalyse the hydrolysis of fatty acid esters bound in the triacylglycerol and release free fatty acids [1]–[2]. Lipases have broad distribution in plants, microorganisms and animal tissues and have many applications in food, diary, detergent and pharmaceutical industries [3].

Porcine pancreatic lipase (PPL) is a secreted glycoprotein composed of a single chain of 449 amino acids, with a molecular weight of 50–52 Kd [4]. It is an endo-lipase, and has high efficiency in catalyzing the hydrolysis of triglycerides and release free fatty acids. In animals the digestion of fat is to a large extent dependent on pancreatic enzymes [5]. The digestibility of fat is of special concern because it has been demonstrated that piglets have a high demand for energy that is not met by the food consumed and consequently, body fat is mobilized to cover the energy requirement [6]. During the postweaning period, the activation, secretion and function of pancreatic digestive enzymes is not yet completely developed, in particular the main lipolytic enzyme lipase and colipase are still underdeveloped [7]. Insufficient production of PPL in early life, in particular at weaning, is a major cause of stress that causes post weaning retardation of growth and leads to substantial economic loss [8]. It is reported that pancreatic-like microbial enzyme (including lipase) supplementation improved the growth of the exocrine pancreatic insufficiency (EPI) in pigs [9], increased piglet activity [10] and enhance piglet gastrointestinal tract development [11]. PPL has also been used for many years as a digestion aid in patients suffering from pancreas dysfunction and malnutrition [12]. Therefore, it may be of practical value to supplement diets for young pigs with exogenous sources of PPL, due to the insufficient production of the endogenous enzyme in young pigs. Commercially available PPL mainly comes from a crude extract preparation of animal pancreases and contain a significant number of other enzymes as contaminants [13]–[14]. The high cost of extraction and purification, limited availability of pig pancreatic tissues, and possible microbial contamination have precluded a large scale application of PPL in animal feed industry. Therefore, it is necessary to develop an efficient yeast expression system for economical and safe supplementation of sufficient amount of the enzyme [14]. Our group and several others have attempted to produce lipase in heterologous systems, but yields are unsatisfactory [15]–[17]. It is reported that heterogeneous protein expression level in *P. pastoris* strongly depends on the biased codon usage [18]–[20], the codon bias of *P. pastoris* may be a factor limiting a high expression of heterologous PPL. Our objective of this study was to develop an efficient expression system in *P. pastoris* to produce a recombinant porcine pancreatic lipase with high yield and activity through partially optimized 5-terminal codon of the porcine pancreatic lipase gene. We also compared the recombinant PPL with the commercial nature form of the enzyme isolated from the pig pancreas. We successfully expressed a rePPL in the *P. pastoris* and found similar enzymatic properties to its native commercial PPL. Our findings suggest a potential approach for producing rePPL for the animal feed industry.

## Materials and Methods

### Ethics statement

The pig protocol for pancreas sample was approved by Animal Care Office of Sichuan Agricultural University, Chengdu, China and the animal experimental procedure was performed according to the guidelines for the care and use of experimental animals established by the Ministry of Agriculture of People's Republic of China. One crossbred (Duroc×Landrace× Yorkshire) male weaned pig (6 weeks of age, 11.4Kg body weight) supplied by Experimental Farm of Animal Nutrition Institute, Sichuan Agricultural University was slaughtered by carbon dioxide, after being anaesthetized with sodium pentobarbital (20 mg/kg body weight), then pancreas sample (about 2 gram) was collected and kept in liquid nitrogen for total RNA extraction and PPL cDNA isolation.

### Strains, plasmids and reagents

The plasmid pPICZαA (Invitrogen, Beijing, China) was used for the production of rePPL proteins and the *P. pastoris* X-33 strain (Invitrogen, Beijing, China) was used as the protein expression host. *E. coli* TOP10 (Invitrogen, Beijing, China) was used for plasmid amplification. The *E. coli* TOP10 strain was grown at 37°C in LB medium and *P. pastoris* X-33 strain was grown in YPD medium at 28–30°C. Plasmid Mini-prep Kit, Gel Extraction Kit and Cycle-pure Kit were purchased from OMEGA (Chengdu, China), Taq DNA polymerase,T4 DNA ligase, pMD18-T vector, restriction enzymes (*Xba* I, *EcoR* I, *Xho* I, *BamH* I, *Sac* I), DL2000 DNA marker, protein marker were purchased from TaKaRa (Dalian, China). Ni-NTA His Binding Resin (GE Healthcare, USA) was used for the purification of recombinant PPL. p-nitrophenyl palmitate (p-NPP) and native commercial PPL was purchased from Sigma (USA). All other chemicals used in this study were of analytical grade and commercially available.

### Codon optimization of porcine pancreatic lipase gene and construction of opPPL/pPICZαA plasmid

A full length cDNA encoding PPL was previous isolated from the pancreas of a Duroc×Landrace×Yorkshire (DLY) crossbred male pig and cloned into pMD18-T by our laboratory [17] and was used for the recombinant PPL/pPICZαA construction in this experiment. The PPL gene fragment with additional *Eco*R I and *Xba* I on 5-and 3-terminal was acquired by PCR amplification using PPL-F and PPL-R primers (Table 1) and cloned into pMD19-T, The PPL/pMD19-T plasmid was sequence confirmed and the resultant cDNA fragments was further cloned into pPICZαA to get the recombinant naPPL/pPICZαA expression plasmids.

**Table 1:** Sequence of primers.

Name	Primer sequence (from 5′ end to 3′ end)
Primers for PPL gene cloning	
PPL-F	AGCTGAATTCATGCTGCTAATCTGGACAC
PPL-R	TTGTTCTAGAAACAGGGGTTGAGGGTG
Primers for o-PPL	
o-PPL-F	ATCTCTCGAGAAAAGAGAGGCTGAAGCT
o-PPL-R	AGCTGGATCCAAACCAGTAATTCTTTCAA
Primers for real time PCR	
RePPL-F′	CTGGGAAAACAAACGGAGTGA
RePPL-R′	CCAACGGGCGAAATTGC
*β-actin*-F	CCAACGTGTGTTTCATTGCA
*β-actin*-R	ATCATGCCCCAAATCAA

**Figure 1:**
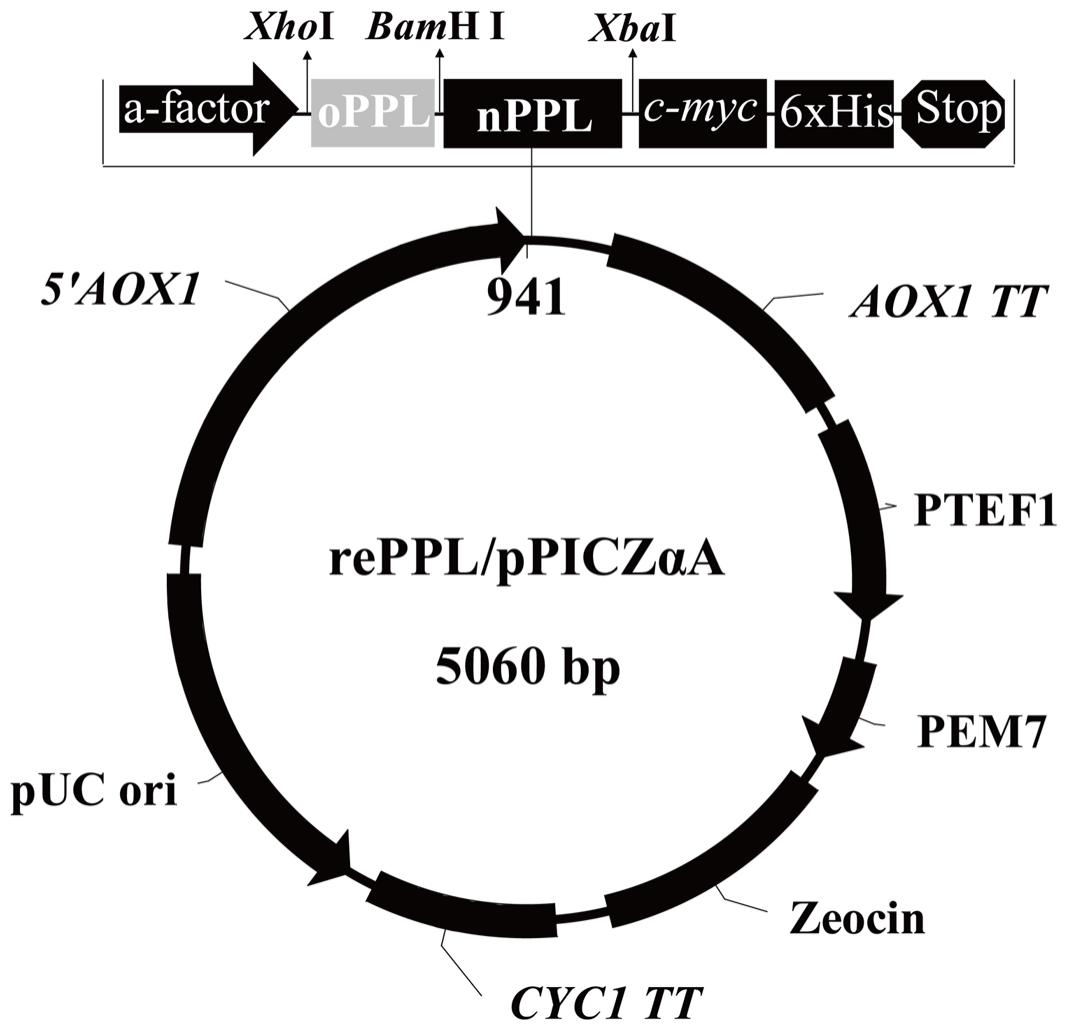
Schematic representation of expression vector opPPL/pPICZαA with *5′AOX1* promoter, fusion partner *c-myc* epitope, and 6×His tag.

The full-length CDS of *PPL* (1398 bp) has two *Bam*H I sites (137 and 575 bp) and a 48 bp signal peptide encoding sequence. The gene was partially codon-optimized according to the strategy showed in Fig. 1: A 537 bp fragment encoding the mature N-terminal of *PPL (oPPL)* was designed according to the codon bias of *P. pastoris* (http://www.kazusa.or.jp/codon) and high frequency codon in *P. pastoris* were used to replace corresponding codon in PPL cDNA fragment. The new cDNA fragment encodes the same amino acid sequence of PPL and contained a second *Bam* H I site after codon optimization (S1 Fig.). The optimized fragment with an additional 28 bp adapter vector sequence which included a *Xho* I site and both *kex*2 and *ste*13 signal cleavage sites (ATCTCTCGAGAAAAGAGAGGCTGAAGCT) on the 5-termianl (total 565 bp) sequence was synthesized and cloned into PUC57 vector (Sangon Biotech, Shanghai) (and named as *oPPL/PUC57*). The optimized 565 bp fragment has an *Xho* I and a *Bam*H I sites on the 5-and 3-terminal ends, separately. These were used for the further full length opPPL gene expression vector construction.

The plasmids oPPL/PUC57 was digested with *Xho* I and *Bam*H I, PPL/pMD19-T was digested with *Xba* I and *Bam*H I, whereas the pPICZαA was digested with *Xho* I and *Xba* I in separate reactions. After 1% agarose gel electrophoresis the 565 bp oPPL fragment, 823 bp nPPL fragment (from the second *Bam*H I site to the 3-terminal of PPL) and the pPICZαA fragment were gel recovered and ligated overnight at 16°C with T4 ligase. The resultant recombinant expression plasmid opPPL/pPICZαA was finally transformed into *E. coli* TOP10 and selected on Low Salt LB agar plates containing 25 µg·mL^−1^ zeocin, 37°C for 16 h. The positive opPPL/pPICZαA plasmid clone was sequence confirmed before further transformed into *P. pastoris* cells.

### Transformation and expression of rePPL in *P. pastoris*

The recombinant plasmid opPPL/pPICZαA and naPPL//pPICZαA were separately transformed into *P. pastoris* X-33 strain by electroporation according to Kim [21]. Single colonies of the transformants were selected for expression according to a protocol of EasySelectTM Pichia Expression Kit (Invitrogen, Beijing, China). The transformant with the highest mRNA expression performance was screened for further comparison of protein production and purification. The *P. pastoris* transformant containing the expression vector pPICZαA without the *PPL* gene insertion was used as the negative control.

### Q-PCR analyses of PPL gene copies and mRNA abundance

DNA of yeast was isolated using yeast DNA Extract Kit (Sangon Biotech,Shanghai,China) according to the manufacturer's instruction. Total RNA extraction and quality control, Q-PCR procedure, and relative gene abundance (DNA or mRNA) quantification were the same as previously described by our group [22]. The genes relative expression were analyzed using Q-PCR (ABI7900HT, Applied biosystem). The primers for *PPL* gene and reference β-actin gene were designed using Primer Express 3.0 (Applied Biosystems, Foster City, CA) and are presented in **Table 1**.

### Purification of recombinant opPPL

The highest mRNA expression recombinant *P. pastoris* clone, named opPPL-13, was grown in 100 mL BMGY medium at 30°C with constant shaking at 250 rpm for about 24 h with OD_600_ reach to 2–5. Cells grown in BMGY were harvested and re-suspended in 100 mL BMMY medium, then 0.5% final concentration of methanol was added to induce protein expression at 30°C with constant shaking at 250 rpm. To maintain induction, 100% methanol was added to the culture to a final concentration of 0.5% every 24 h. After 96 h of methanol induction, the fermentation broth was centrifuged at 14,000 g at 4°C for 10 min to remove cells. The supernatant was added with 0.5 mM NaCl and adjusted to pH 7.4, followed by filtration through a 0.45 µm filter. The supernatant was then applied to a Ni Sepharose (GE Healthcare, USA) affinity column (Bio-Rad, Richmond, CA, USA) pre-equilibrated with a binding buffer (20 mM NaH_2_PO_4_, pH 7.4, 500 mM NaCl, 20 mM imidazole). After the column was washed with binding buffer to remove the unbinding proteins, the opPPL was eluted with elution buffer (20 mM NaH_2_PO_4_, pH 7.4, 500 mM NaCl, 500 mM imidazole). Fractions containing opPPL were pooled and further desalted with a 10 kDa molecular cutoff concentration tube (Millipore) using 20 mM PBS buffer (pH 7.4). The purified opPPL protein and purchased commercial PPL were stored at −20°C for subsequent analysis. Protein samples were separated on 12% SDS–polyacrylamide gel electrophoresis (SDS–PAGE). The protein bands were visualized by staining with coomasse brilliant blue. Protein concentration was determined by the Bradford method [23].

### Yield comparison of opPPL and naPPL

The highest mRNA expression opPPL and naPPL transformant were induced by methanol for PPL expression as the same condition described above (n = 3). After 96 h of methanol induction, the supernatant were harvested and volume were calculated, then 10 ml supernatant of each samples were further desalted with a 10 kDa molecular cutoff concentration tube (Millipore) using 20 mM PBS buffer (pH 7.4), the crude recombinant PPL samples were collected and stored at −20°C before use. The recombinant PPL yields were determined by comparison of the PPL band on SDS-PAGE gel against BSA standard protein bands (1 µg and 2 µg/well) using Image Lab^TM^ Software version 4.1 (Bio-rad, Shanghai China). Also the enzyme activities of samples were assayed and total enzyme activities were compared.

### Assays of PPL

PPL activity was quantitatively assayed using p-NPP as substrate. This assay was performed as method described by Winkler and Stuckman [24] with some modifications. A stock solution of p-NPP was freshly prepared in 2-propanol at a concentration of 10 mM. Then, 900 µl of 1∶9 dilution of the substrate stock solution A in solution B (0.1% (w/v) gum arabic, 0.4% (v/v) Triton X-100 in distilled water with 50 µl of appropriate buffer were pre-incubated for 5 min at 40°C before adding 50 µl of enzyme sample(40 ng PPL protein). This mixture was incubated at 40°C for 10 min, and the reaction was terminated by addition of 2 ml of 0.2 M Na_2_CO_3_ solution. Released p-NP was immediately determined by measuring the absorbance at 410 nm in a Beckman spectrophotometer (Model DU 800, USA). Appropriate blanks were used to subtract the absorbance corresponding to the reaction mixture other than that produced by the specific hydrolysis of p-NPP. The molar extinction coefficient of p-NP (*ε*_410 nm_ = 16,900 M^−1^ cm^−1^) was estimated from the absorbance of standard solutions of p-NP. One international unit of lipase activity was expressed as the amount of enzyme liberating 1 µM of p-NP per minute under the conditions of the assay [25].

### Characterization of opPPL

The purified opPPL was used for further enzyme characterization. To study pH optimum, temperature optimum and thermostability and substrate specificity, the reaction mixture remained the same as above except for the buffers and the temperature of the enzymatic reaction. The optimal pH profile of opPPL was assayed at 40°C using citrate buffer (pH 4.0–6.0), phosphate buffer (pH 7.0), Tris-HCl buffer (pH 8.0–9.0) and glycine-NaOH buffer (10.0–11.0). The optimal temperature of opPPL was determined using the Tris-HCl buffer (pH 8.0) from 20 to 80°C. The thermal stability of opPPL was determined by the residual activity after the enzyme was incubated in the Tris-HCl buffer (pH 8.0) at 30, 40, 50 and 60°C for 10, 20, 30 and 60 min respectively. The kinetic parameters (*K_m_* and *V_max_*) for purified opPPL were calculated at pH 8.0 and at 40°C from initial velocities in the 0.02–0.1 mM range of p-NPP using the Lineweaver-Burk method [26]. To test how opPPL might function under the intestinal conditions, the purified enzyme was incubated with various concentration of Chloride metal ions (Zn^2+^, Cu^2+^, Fe^3+^, Ca^2+^) in the phosphate buffer at 37°C for 10 min and the activity change against the untreated control was determined. The enzyme properties of commercial Sigma enzyme(named commercial PPL)were also compared. Three replicate were conducted in each assay and data were shown with mean±SE(n = 3).

## Results

### Codon optimization, expression and purification of opPPL in *P. pastoris*

**Figure 2:**
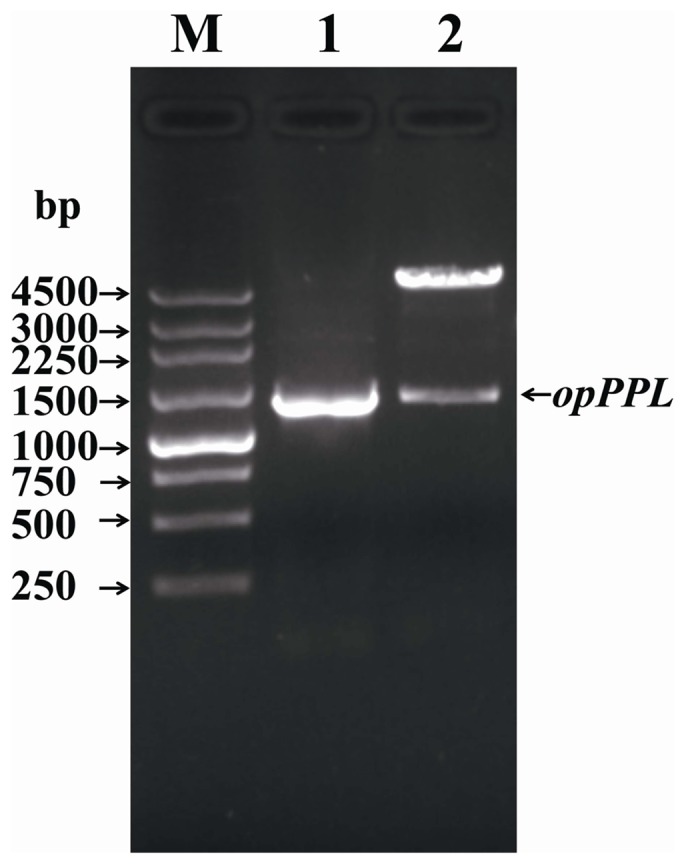
Construction of opPPL/pPICZαA. Lane 1, PCR products of the opPPL cDNA; Lane 2, *Xho* I and *Xba* I–digested opPPL/pPICZαA; Lane M, molecular Marker.

**Figure 3:**
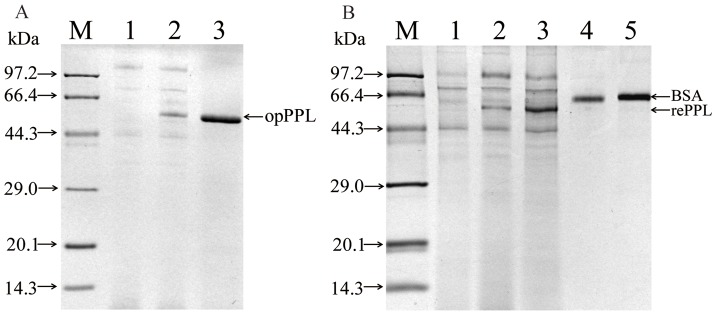
SDS-PAGE identification of opPPL expressed in *P.pastoris* and comparison of recombination PPL yield. (A) Identification of opPPL expressed in *P.pastoris* by 12% SDS-PAGE. Lane M, molecular markers; lane 1, crude supernatant from control pPICZαA; lane 2, crude supernatant from opPPL-13; lane 3, purified opPPL. (B) Comparsion of recombination PPL yields. Lane M, molecular markers; lane 1, crude supernatant from control pPICZαA(10 µl); lane 2, crude supernatant from naPPL/pPICZαA(10 µl); lane 3, crude supernatant from opPPL-13(10 µl); Lane 4, BSA standard protein bands (1 µg); Lane5,BSA standard protein bands (2 µg).

A full length cDNA encoding PPL was previously isolated from the pancreas of pig by our laboratory and was used for the rePPL expression vector construction in the yeast system. A 537 bp 5-terminal encoding fragment of the mature PPL was codon-optimized according to the codon bias of *P. pastoris* (S1 Fig.) and the partial codon optimized *PPL* (*opPPL*) were cloned into pPICZαA according to the strategy described in Fig. 1. After the cloned expression vector opPPL/pPICZαA was digested with *Xho* I and *Xba* I, a ca 1,500 bp target gene band and a 3,600 bp expression vector band were shown on the 1% agarose gel (Fig. 2, lane2). Sequencing of the cloned opPPL/pPICZαA showed it still encoding the same amino acid sequence of the native one after codon optimization (S2 Fig.). The opPPL/pPICZαA was transformed into *P.pastoris* X-33 and the highest mRNA expression transformant was screened by Q-PCR and use for opPPL expression (S3 Fig.). After the *P. pastoris* X-33 transformant was induced by 0.5% methanol for 96 h, the target protein was purified by Ni Sepharose affinity chromatography. The purified opPPL showed a single band on 12% SDS-PAGE gel with a molecular size of approximately 52 kDa (Fig. 3A, lane 3).

### Comparison of recombinant PPL yields, PPL plasmid copies and relative mRNA profiles

**Figure 4:**
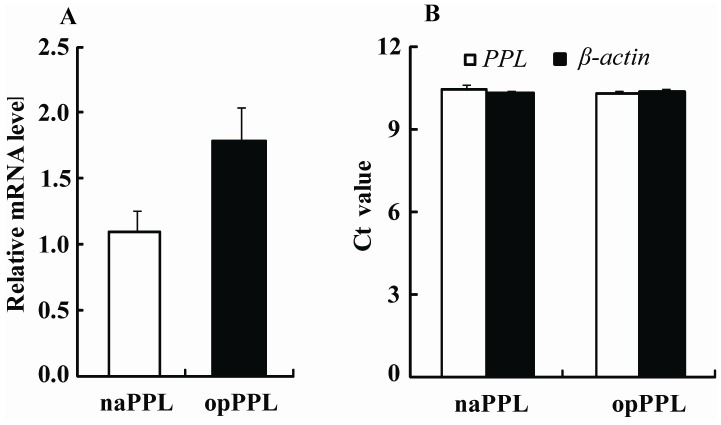
Comparison of the target gene relative mRNA profiles (A) and gene copies numbers (B) between the *P.pastoris* opPPL//pPICZαA and naPPL/pPICZαA.

After 96 h fermentation induced by methanol, the culture supernatants were harvested and applied for SDS-PAGE analysis. The codon optimized recombination protein showed darker and stronger band on the gel (Fig. 3B, lane 3) than that from non-optimized one (naPPL). (Fig. 3B, lane 2). The yield of the opPPL was calculated to be 146 mg.L^−1^, about 4-fold higher compared with 36 mg.L^−1^ of naPPL. Also total enzyme activity was increased from 367 IU.L^−1^ to 1900 IU.L^−1^ after codon optimization. Take *β-acin* as reference gene, the real time q-PCR for DNA samples shows the target *PPL* gene shares the same Ct value in both opPPL/pPICZαA and naPPL/pPICZαA strains and its share the same gene/*β-acin* ratio (Fig. 4B), which suggests the same *PPL* gene copies between the two transformants. As shown in Fig. 4A, after 96 h induction by methanol, the opPPL/pPICZαA strain showed about 1.6 fold higher mRNA profile than that of the *naPPL/pPICZαA strain*.

### Characterization of opPPL

**Figure 5:**
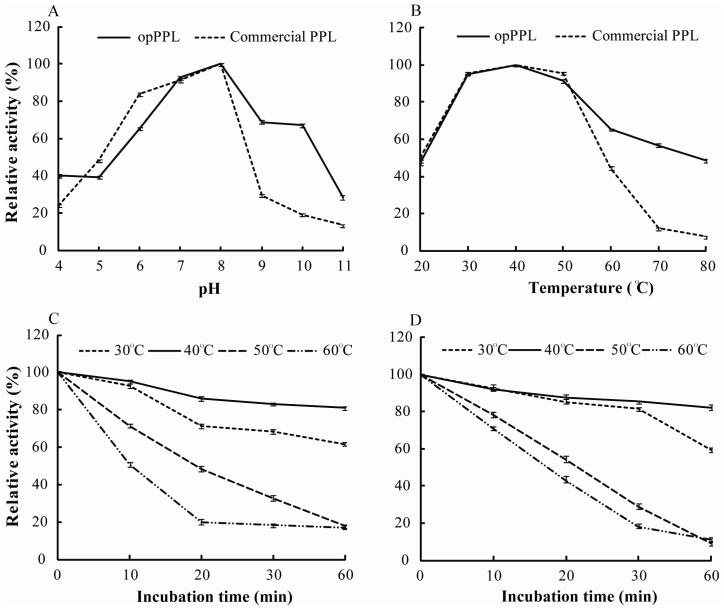
Comparison of opPPL and commercial PPL activity. (A) Effect of pH on the activity of opPPL and commercial PPL. (B) Effect of temperature on the activity of opPPL and commercial PPL.(C) The thermostability of commercial PPL. (D) The thermostability of purified opPPL. These assays were performed as described in Materials and methods using 10.0 mM p-NPP as substrate (n = 3). The maximum value was taken as 100%.

**Figure 6:**
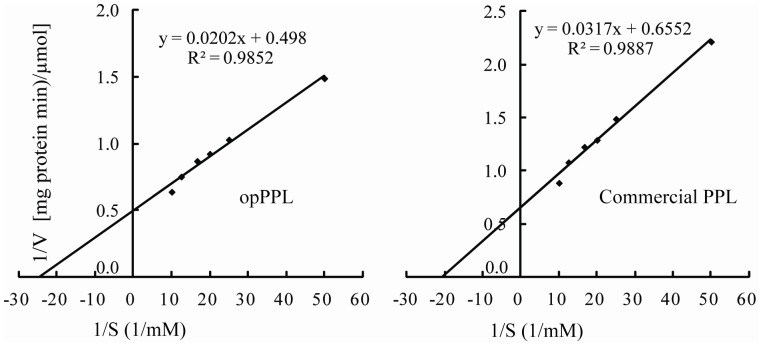
The *K_m_* value of opPPL and commercial PPL against p-NPP determined by Lineweaver-Burk method. (A) The *K_m_* value of opPPL. (B) The *K_m_* value of commercial PPL.

**Figure 7:**
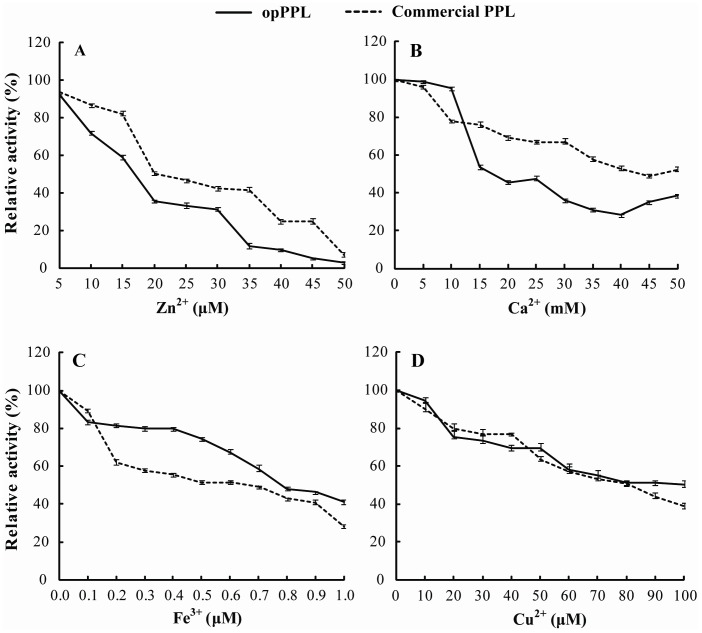
Effect of metal ions on opPPL and commercial PPL activity. (A),(B),(C) and (D) represent the effect of Zn^2+^, Ca^2+^, Fe^3+^ and Cu^2+^ on the activity of opPPL and commercial PPL, respectively. y means relative enzyme activity; x means concentration of the metal ions; These assays were performed as described in Materials and methods using 10.0 mM p-NPP as substrate(n = 3). The maximum value was taken as 100%.

As shown in Fig. 5, opPPL and the commercial native form of PPL enzyme shared similar pH-activity and temperature-activity profiles. The optimal pH of opPPL was 8.0, while the enzyme maintained>50% activity between pH 6.0 and 8.0, it dropped sharply when pH beyond 8.0 (Fig. 5A). The opPPL exhibited strong activity from 20°C to 50°C, with an optimum temperature of about 40°C, and the activity decreased sharply at temperatures>50°C (Fig. 5B). The thermal stability of the purified opPPL and commercial PPL essentially paralleled the effect of temperature on the activity in that pre-incubation at 30°C or 40°C for 30 min had little impact on the activity while pre-incubation at 50°C caused the activity to drop in proportion to time of incubation, and as little as 20 min incubation at>60°C caused a sharp loss of activity (Fig. 5C, 5D). The purified opPPL showed a *K_m_* for p-NPP as 0.041 mM and *V_max_* as 2.008 µmol. mg protein −1. min−1, whereas the commercial form of PPL had a *K_m_* of 0.048 mM and *V_max_* as 1.526 µmol. mg protein −1.min−1 (Fig. 6). The effect of metal ions on activity of the opPPL and commercial enzyme were compared and the activity of opPPL and commercial PPL enzyme manifested a dose-dependent decrease (P<0.05) by incubating with Ca2+, Zn2+, Cu2+, or Fe3+ (Fig. 7).

## Discussion

We successfully partially optimized porcine pancreatic lipase gene and effectively increased it expression in *P.pastoris* in the present study. The α-factor signal peptide in the yeast expression vector was effective in guiding the secretion of the recombined PPL into the culture broth, which may facilitate the production of lipase for direct industrial application without complicated purification procedures [27]. After 4 days of growth in yeast, the corresponding amount of opPPL reached about 146 mg.L^−1^ of culture medium. This production rate is higher in comparison with other production levels of various lipases expressed in *P.pastoris*. For comparison, the production rate of the turkey pancreatic lipase is about 15 mg.L^−1^ of culture medium after 3 days of methanol induction [28]. The human pancreatic lipase-related protein 2 has a productivity 40 mg.L^−1^ under the control of the AOX1 promoter and about 4 mg.L^−1^ under the control of the constitutive GAP promoter [29]. Fugal resources (*C.rugosa*) Lip4 lipase showed a 0.1 g.L^−1^ production in *P.pastoris* system[30]–[31]. As the PPL gene contains many codons that are rarely or less frequently used in *P.pastoris*, optimizing the usage of these codons may improves rePPL protein production by the yeast host. In this study, partial optimization of the N-terminal 537 bp codons of PPL greatly increased the opPPL yield compared with those native PPL tranformant (146 mg.L^−1^vs 36 mg.L^−1^), which is almost a three-fold improvement compared with our lab's previous 43 mg.L^−1^ yield [17]. In addition, the total enzyme activity against p-NPP greatly improved (1900 IU.L^−1^vs 367 IU.L^−1^). Our results were much consistent with previous studies demonstrated that the codon optimization echnique greatly increases (about 1- to 10-fold) the foreign proteins expression in *P. pastoris* [32]–[33]. The increased opPPL yields may be partly ascribed to increased protein translational efficiency because there was only a 1.6 fold higher mRNA expression after codon optimization (Fig. 4A). The opPPL protein was>25% of the total proteins secreted into the culture medium (Fig. 3A, lane 2), which indicates a potential for industrial production, purification and application.

However, the enzyme yield (146 mg.L^−1^) was lower than that of some fungal lipases in *P.pastoris* such as *Yarrowia lipolytica* (0.63 g.L^−1^) [34] and *Mutant Nippostrogglus brasiliensis* Acetylcholinestrease (2 g.L^−1^) [35]. Because the inducible *P. pastoris* expression system is able lead to production of heterologous proteins intracellularly and extracellularly up to 3 g.L^−1^ and 12 g.L^−1^, respectively [36]–[37], therefore, future research will be needed to enhance the opPPL yield. In our study, the PPL gene optimized strain has the same plasmid copies with the non-optimized one (Fig. 4B). Therefore, increasing number of copies of the expression plasmid may increase the recombinant protein expression [38]. In addition, the protein production efficiency may be enhanced by optimizing fermentation conditions including medium pH, temperature, and methanol concentration [14].

In this study, *Xho* I site was used to construct the opPPL/pPICZαA vector, thus the expressed opPPL has the same N-terminus amino acid compared with the native protein, and a his-tag attached in the C-terminus of the opPPL was designed to favor protein purification. The over-expressed opPPL in *P. pastoris* shared similar enzymatic properties to those of the commercial native form as determined in the present study and (or) reported by previous researchers. Specifically, the opPPL and the native form of PPL (Sigma) had *k_m_* for p-NPP: 0.041 and 0.048 mM, respectively (Fig. 5). The *V_max_* (2.008 µmol. mg protein ^−1^. min^−1^), optimal pH (8.0), and optimal temperature (40°C) of opPPL estimated in the present study was similar to those reported for the native enzyme [39]. These similarities indicate that the heterologous expression of opPPL in the *Pichia* yeast system did not alter its enzymatic property or function. The opPPL exhibited strong activity at the physiological pH and temperature condition in duodenums of piglets, which may indicate the advantage of opPPL over lipases of fugal or bacterial origins in feed supplementation for young piglets. Practically, the recombinant lipase may be supplemented in diets for young pigs to replace or supplement the endogenous enzyme in the gut. It is remarkable that the 23 amino acid his-tag in the C-terminus of the opPPL protein exerted no negative impact on the enzyme activity or catalytic function. This flexibility may allow genetic or molecular manipulations to favor enzyme fermentation and corresponding protein product purification.

The recombinant opPPL protein was also tested for thermostability and responses to divalent metals, two most relevant measures for its application in animal feeding. Because a large portion of feed for monogastric animals such as pigs is pelleted, exogenous enzymes as feed additive must be tolerant to inactivation by heat and steam during the pelleting process [40]. Although the purified opPPL was fairly stable at 30°C and 40°C, substantial activity was lost after incubation at to 50 or 60°C. Thus, thermostability of this enzyme needs improving by protein engineering [41] for animal feed industrial applications. In the pig digesta, free ion concentrations (µM) were reported as follows: Cu = 5.5–31.6, Fe = 3–29, Zn = 44–132, Ca = 1,100–5,400[42]. Based on the activity response curves of opPPL to different ions shown in Fig.6, the pig digesta concentrations of Cu or Fe should have little inhibition of opPPL activity. It has been suggested that PPL may bind Fe with the -SH groups of cysteine residues in the protein chain [43]. However, Zn in the pig digesta was within the range of concentration that may result in approximately 40–50% inhibition of opPPL activity. This inhibition by Zn may be attributed to a binding to the catalytic residues [44]. Therefore, it is necessary to improve resistance of the enzyme to inhibition by Zn and (or) control dietary Zn concentration for a satisfactory efficacy of the supplemental opPPL in piglet.

In summary, we have successfully partially codon optimized the porcine pancreatic lipase gene and proved greatly increasing the expression of the gene in *P. pastoris* into an extracellular, functional enzyme. Our biochemical characterization of the recombinant enzyme showed it has similar properties to the commercial lipase isolated from porcine pancreases. This indicates its potential application as an exogenous enzyme supplement in animal feed.

## Supporting Information

S1 Fig **The optimized** ***opPPL*** **gene sequence according to PPL amino acids and codon bias of** ***Pichia pastoris*** **(without the signal peptide).**
(TIF)

S2 Fig **Deduced amino acid sequence of the constructed opPPL/pPICZαA.**
(TIF)

S3 Fig **Screen of high opPPL mRNA expression transformants by Q-PCR.** 25 positive *P.pastoris* opPPL/pPICZαA clones were separately induced with 0.5% final concentration of methanol in 100 ml medium for 72 h according to material and method, then total RNA were extracted for relative target gene mRNA profiles comparison using real time Q-PCR. The values were present with mean±SEM.
(TIF)
