# Supplementary figures and images for: Partial Optimization of the 5-Terminal Codon Increased a Recombination Porcine Pancreatic Lipase (opPPL) Expression in Pichia pastoris
Source: PLoS One. 2014 Dec 29;9(12):e114385. doi: 10.1371/journal.pone.0114385 (PMC4278863; doi:10.1371/journal.pone.0114385)

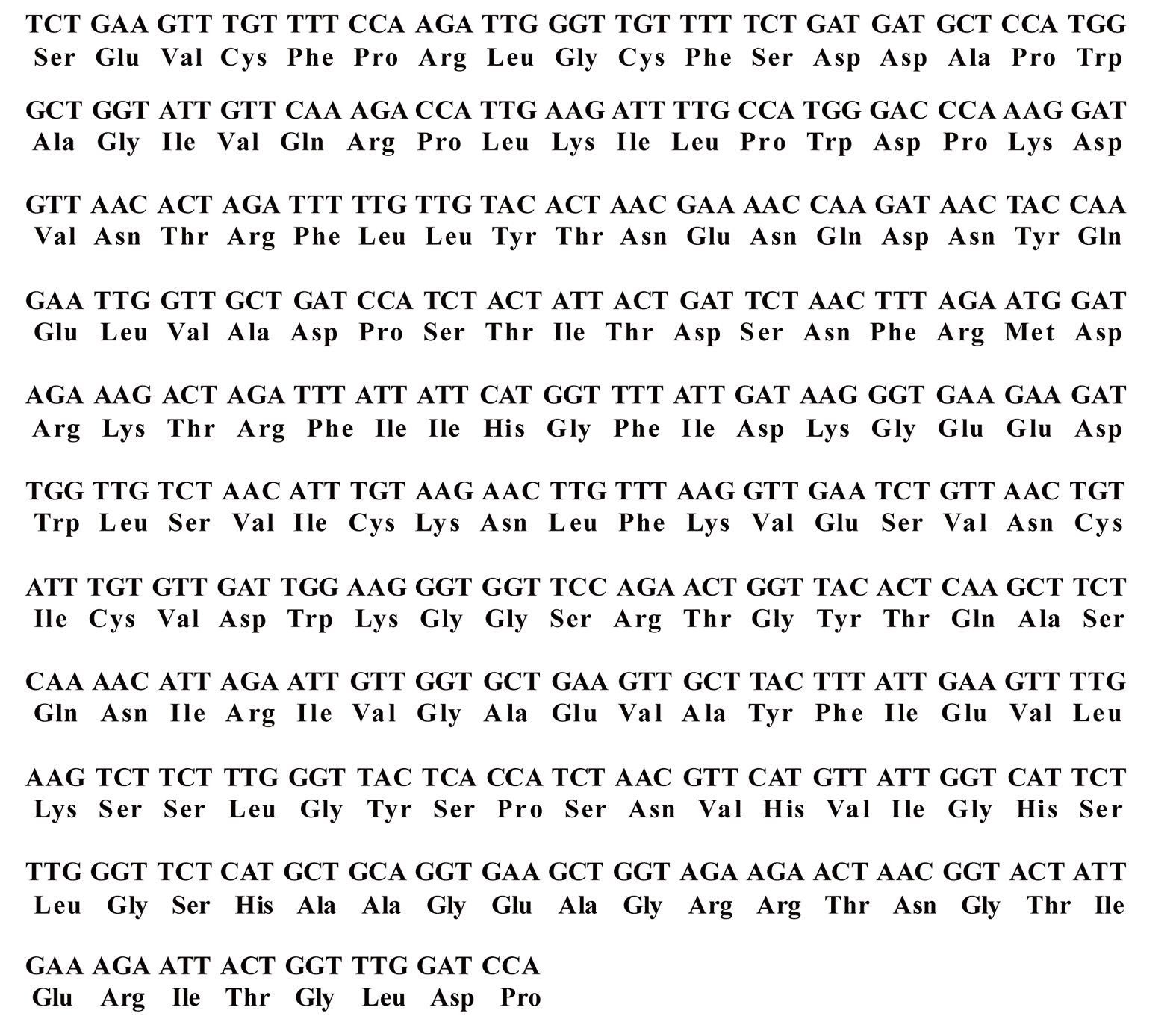

Supplement: S1 Fig — The optimized opPPL gene sequence according to PPL amino acids and codon bias of Pichia pastoris (without the signal peptide). (TIF) [file pone.0114385.s001.tif]

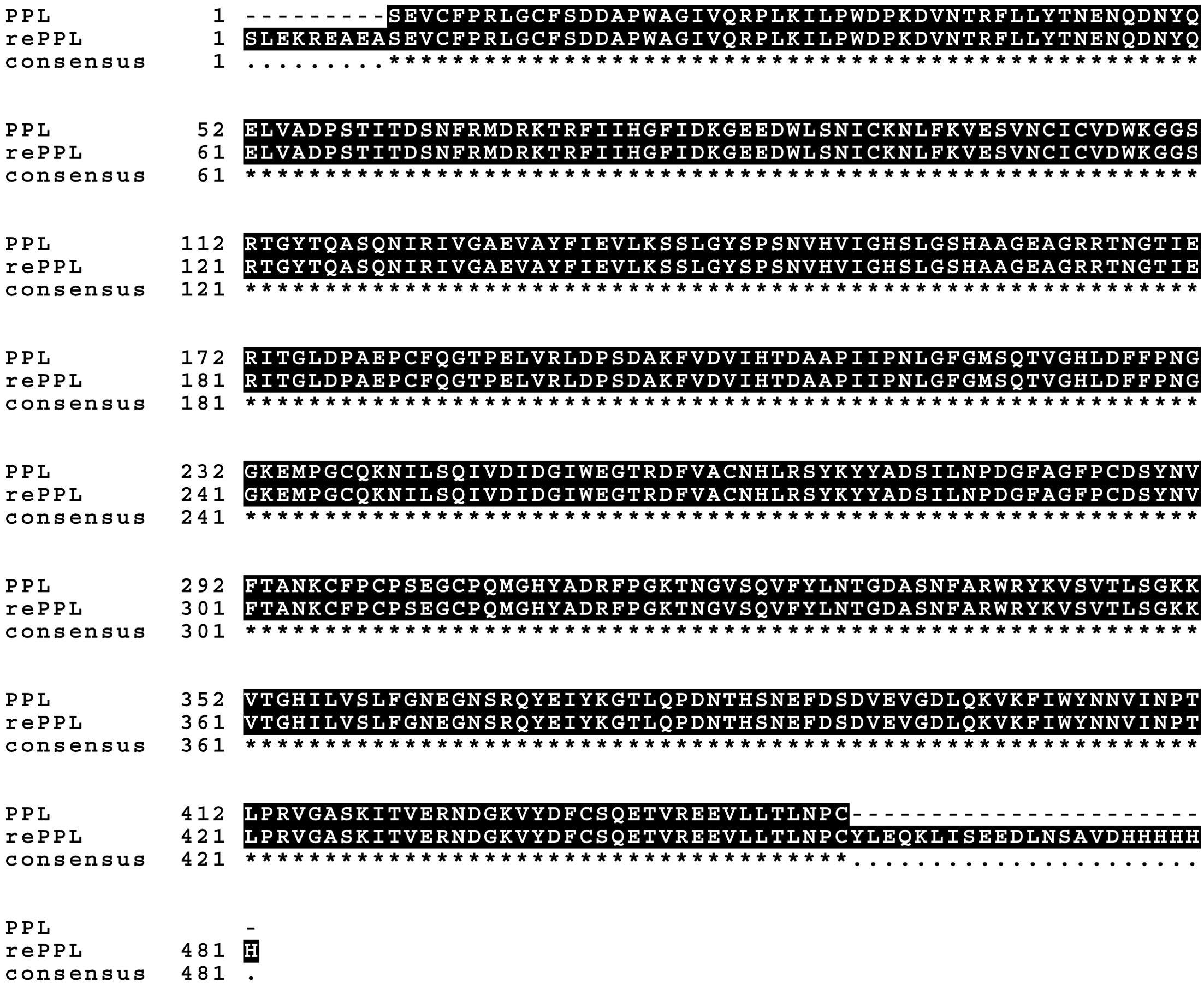

Supplement: S2 Fig — Deduced amino acid sequence of the constructed opPPL/pPICZαA. (TIF) [file pone.0114385.s002.tif]

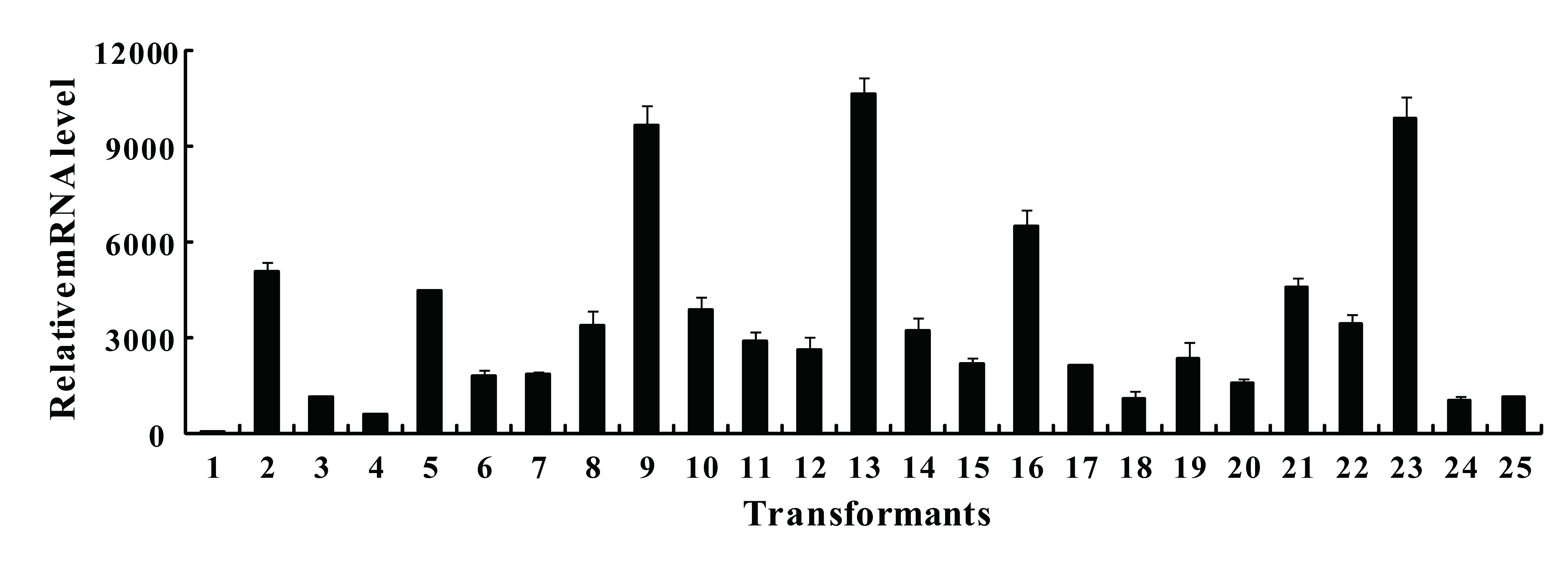

Supplement: S3 Fig — Screen of high opPPL mRNA expression transformants by Q-PCR. 25 positive P.pastoris opPPL/pPICZαA clones were separately induced with 0.5% final concentration of methanol in 100 ml medium for 72 h according to material and method, then total RNA were extracted for relative target gene mRNA profiles comparison using real time Q-PCR. The values were present with mean±SEM. (TIF) [file pone.0114385.s003.tif]
